# DRP1 deficiency induces mitochondrial dysfunction and oxidative stress-mediated apoptosis during porcine oocyte maturation

**DOI:** 10.1186/s40104-020-00489-4

**Published:** 2020-08-05

**Authors:** Haolin Zhang, Zhennan Pan, Jiaqian Ju, Chunhua Xing, Xiaohan Li, Mengmeng Shan, Shaochen Sun

**Affiliations:** College of Animal Science and Technology, Nanjing Agricultural University, Nanjing, 210095 China

**Keywords:** Apoptosis, Meiosis, Mitochondria, Oocyte, Oxidative stress

## Abstract

**Background:**

Environmental pollution induces oxidative stress and apoptosis in mammalian oocytes, which can cause defects in reproduction; however, the molecular regulation of oxidative stress in oocytes is still largely unknown. In the present study, we identified that dynamin-related protein 1 (DRP1) is an important molecule regulating oocyte mitochondrial function and preventing oxidative stress/apoptosis. DRP1 is a member of the dynamin GTPase superfamily localized at the mitochondrial-endoplasmic reticulum interaction site, where it regulates the fission of mitochondria and other related cellular processes.

**Results:**

Our results show that DRP1 was stably expressed during different stages of porcine oocyte meiosis, and might have a potential relationship with mitochondria as it exhibited similar localization. Loss of DRP1 activity caused failed porcine oocyte maturation and cumulus cell expansion, as well as defects in polar body extrusion. Further analysis indicated that a DRP1 deficiency caused mitochondrial dysfunction and induced oxidative stress, which was confirmed by increased reactive oxygen species levels. Moreover, the incidence of early apoptosis increased as detected by positive Annexin-V signaling.

**Conclusions:**

Taken together, our results indicate that DRP1 is essential for porcine oocyte maturation and that a DRP1 deficiency could induce mitochondrial dysfunction, oxidative stress, and apoptosis.

## Background

Mammalian oocytes undergo two consecutive rounds of meiotic division and generate a large haploid egg and two small polar bodies [1]. The majority of the oocyte cytoplasm is retained during this process, which contains most of the organelles including mitochondria, providing sufficient storage materials and dynamophore (energy carrier) for early embryonic development after fertilization [2]. Mitochondria are specialized organelles within the cytoplasm of all eukaryotes and they are the center of cellular and metabolic homeostasis for diverse physiological processes [3]. Mitochondria form a functional reticulum whose steady-state morphology is regulated by dynamic fission, fusion, and motility events [4]. Mitochondrial fusion is mediated through the action of at least three GTPases, such as mitofusin 1, mitofusin 2 (MFN1, MFN2), and optic atrophy 1 (OPA1) [4], while dynamin-related protein 1 (DRP1) and its mitochondrial receptors including the 49 and 51 kDa mitochondrial dynamics protein paralogues and mitochondrial fission factor are required for mitochondrial fission in mammals [5]. Disordered mitochondrial dynamics alters metabolism, proliferation, apoptosis, and mitophagy, contributing to human diseases, such as neurodegenerative syndromes, pulmonary arterial hypertension, cancer, and ischemia/reperfusion injury [6].

Dynamins are a large GTPase superfamily that includes classical dynamins, DRP1, OPA1, Mx proteins, mitofusins and guanylate-binding proteins/atlastins in eukaryotic cells, which are involved in the budding of transport vesicles, division of organelles, cytokinesis, and pathogen resistance [7]. DRP1 contains a dynamin-like central domain and a C-terminal GTPase effector domain in addition to its N-terminal GTPase [8]. DRP1 is generally distributed in the cytoplasm and mitochondria [9]. Inactive DRP1 is normally found in the cytosol, while activated DRP1 is translocated to the mitochondrial outer membrane (MOM) [10].

DRP1 mediates mitochondrial fission: small DRP1 oligomers self-assemble into larger multimeric structures at the MOM, where they mediate mitochondrial division through a GTP-dependent conformational change [11]. A DRP1 mutation causes significant aggregation and tubulation of mitochondria [12]. Moreover, DRP1 is involved in various important cellular processes related to apoptosis and necrosis [13]. There is increased recruitment of DRP1 to mitochondria during programmed cell death (apoptosis), with the increase of mitochondrial fission and the decrease of fragment fusion to mitochondria [11]. DRP1 is highly expressed in lung cancer tissues, indicating that DRP1 is involved in the regulation of cancer cell apoptosis [9]. Moreover, mice with tissue-specific depletion of DRP1 exhibit severe damage in various tissues, such as neurodegeneration in neuron-specific DRP1 knockout mice [14, 15]. Oocyte-specific depletion of DRP1 induces female fertility and ovulation defects in mice. These oocytes have an abnormal mitochondrial distribution, and multi-organelle aggregation. Moreover, oocytes from aged mice display reduced DRP1-dependent mitochondrial fission and defective organelle rearrangement [16].

Oocyte maturation in pigs is extended with a higher potential for apoptosis compared to mice. Moreover, the mitochondrial distribution pattern and number are also different with mouse oocytes due to localization on the chromosome and the size of the oocytes. Although the functions of DRP1 have been interpreted in multiple models, whether DRP1 expresses and has roles during porcine oocyte maturation remains unclear. In the present study, we explored the expression and localization of DRP1 in porcine oocytes. We also inhibited DRP1 activity to examine its roles in mitochondrial function, reactive oxygen species (ROS) production, and induction of apoptosis during porcine oocyte maturation. Our results provide evidence for the critical roles of DRP1 monitoring oxidative stress and apoptosis in porcine oocytes.

## Methods and material

### Antibodies and chemicals

Mouse polyclonal anti-DRP1 antibody was purchased from Abcam (Cambridge, UK). Hoechst 33342 was purchased from Sigma (St. Louis, MO, USA). Goat anti-rabbit IgG/FITC and goat anti-rabbit IgG/TRITC were purchased from Zhongshan Golden Bridge Biotechnology (Beijing, China). Basic maturation culture medium was tissue culture medium (TCM-199) (Sigma). Mito-Tracker Red CMRos (1:500, Cat# M7521, Invitrogen, Eugene, OR, USA) was used to detect the distribution of mitochondria with an ultimate density of 2 μmol/L. The DCFH diacetate (DCFHDA) kit (Beyotime, Beijing, China) was purchased to determine the ROS levels in living oocytes. Annexin-V-FITC (Yeasen Biotech Co., Ltd., Shanghai, China) was used to determine apoptosis levels. Mdivi-1 was purchased from MedChemExpress (Shanghai, China).

### Oocyte harvest and *in vitro* maturation

All animal manipulations were conducted in accordance with the guidelines of the Animal Research Committee of Nanjing Agricultural University, China. This study was approved by the Animal Research Committee, Nanjing Agricultural University, China. Ovaries were collected from prepubertal gilts at a local slaughterhouse, placed in 0.9% physiological saline in a thermos bottle and transported to our laboratory within 2 h. After washing twice with sterile Dulbecco's Phosphate Buffered Saline (DPBS) , cumulus-oocyte complexes (COCs) were aspirated from 3 to 6 mm antral follicles using a 20-gauge needle attached to a 10-mL disposable syringe. Oocytes with intact and compact cumulus cells and a uniform ooplasm were selected for study. The medium used for the maturation culture was improved TCM-199 supplemented with 75 μg/mL penicillin, 50 μg/mL streptomycin, 0.5 μg/mL follicle stimulating hormone, 0.5 μg/mL luteinizing hormone, 10 ng/mL epidermal growth factor (EGF), and 0.57 mmol/L cysteine. Oocytes were cultured in 500 μL of maturation medium covered with a thin layer of mineral oil at 38.5 °C for 27 or 44 h in a humidified atmosphere of 5% CO_2_ in a four-well culture dish (Nunc, Roskilde, Denmark). After different culture times, COCs were treated with 0.1% hyaluronidase (in TCM-199) for 5 min at 38.5 °C. The surrounding cumulus cells were stripped by gentle pipetting. After 3–4 rinses, the denuded oocytes were collected for subsequent analysis. We used an inverted microscope (× 200) to check the polar body, and we rotated the oocytes to ensure proper judgment.

### Mdivi-1 treatment

Mdivi-1 is a quinazolinone originally described as a selective inhibitor of Drp1, and reported to inhibit mitochondrial fission by blocking the GTP-induced conformational changes that are necessary to promote self-assembly [17]. Therefore, we used Mdivi-1 to inhibit DRP1 activity and study the effect of DRP1 deficiency on porcine oocyte maturation. Mdivi-1 was dissolved in DMSO to 150 mmol/L for storage, and then diluted to 100 and 300 μmol/L per well in a final volume of 500 μL of *in vitro* maturation medium, with < 0.2% DMSO in the medium. The oocytes were treated with Mdivi-1 from the beginning of culture at the germinal vesicle (GV) stage. We cultured the oocytes for 27 and 44 h to obtain metaphase 1 (MI) and metaphase II (MII) stage oocytes, respectively.

### RNA isolation and quantitative real-time polymerase chain reaction (RT-PCR)

Porcine COCs were matured *in vitro* for 27 h, and the oocytes in the control and Mdivi-1 groups were collected. Total RNA was extracted from approximately 40 oocytes with a Dynabead mRNA DIRECT kit (Invitrogen Dynal, Oslo, Norway), according to the manufacturer’s instructions. First-strand cDNA was synthesized conducted with a cDNA synthesis kit following the manufacturer’s instructions (Takara Biomedical Technology, Dalian, China.) and stored at − 20 °C until analysis. The levels of relevant mRNAs were determined by quantitative RT-PCR using a FastStart Universal SYBR Green Master (Rox; Roche Applied Science, Mannheim, Germany) with the One plus Real-Time PCR System (Applied Biosystems, Life Technologies, Carlsbad, CA, USA), Gene expression levels were analyzed using the 2 ^−^ΔΔCt method after the melting-curve analysis was completed. The expression levels of the target genes were normalized to the expression level of GAPDH in each sample. The primers were: superoxide dismutase (*SOD)1*, F: 5΄-ATC AAG AGA GGC ACG TTG GA-3΄; R: 5΄-TCT GCC CAA GTC ATC TGG TT-3΄. *SOD2*, F: 5΄-TCA AGG AGA AGT TGA CCG CT-3΄; R: 5΄-AGG TAA TAC GCA TGC TCC CA-3΄. *CAT*, F: 5΄- AGA TGG ACA CAG GCA CAT GA-3΄; R: 5΄-CCG GAT GCC ATA GTC AGG AT-3΄. Glutathione peroxidase (*GPx)*, F: 5΄-CAC CCA GAT GAA TGA GCT GC-3΄; R: 5΄-CAT GAA GTT GGG CTC GAA CC-3΄. *BCL-xL*, F: 5΄-ACT GAA TCA GAA GCG GAA AC-3΄; R: 5΄-AAA GCT CTG ATA CGC TGT CC-3΄. *BAX*, F: 5΄-CGC TGG ACT TCC TTC GAG AT-3΄, R: 5΄-CGA TCT TGG TGA AGT ACT C-3΄. *Caspase3*, F: 5΄-GAG GCA GAC TTC TTG TAT GC-3΄; R: 5΄-CAT GGA CAC AAT ACA TGG AA-3΄. *GAPDH*, F: 5΄-AAG TTC CAC GGC ACA GTC AAG-3΄; R: 5΄-CAC CAG CAT CAC CCC ATT T-3΄.

### ROS assay

To analyze the levels of intracellular ROS in living oocytes, a Reactive Oxygen Species Assay Kit was applied to detect ROS as a green fluorescent DCFH-DA signal. Briefly, 20–30 denuded oocytes from each treatment group were incubated with 10 μmol/L DCFH-DA (in D-PBS) for 30 min at 38.5 °C. After washing three times in D-PBS containing 0.1% bovine serum albumin (BSA), the samples were mounted on glass slides, and the fluorescence intensity of each oocyte was measured under a Zeiss LSM 700 META confocal system (Carl Zeiss Inc., Jena, Germany). The fluorescence pixel intensities were analyzed using ImageJ software (version 1.50; National Institutes of Health, Bethesda, MD, USA).

### Annexin-V analysis

To detect the externalization of phosphatidylserine in early apoptotic oocytes, Annexin V-FITC staining was performed using an Annexin V-FITC/EGFP Apoptosis Detection Kit (Vazyme Biotech Co., Ltd., Nanjing, China) according to the manufacturer’s instructions. Briefly, 20–30 MI oocytes from each group were incubated in 100 μL binding buffer containing 10 μL of Annexin V-FITC for 30 min in the dark after washing twice in TCM-199. The oocytes were transferred to a TCM-199 drop in living cell culture dishes. The Annexin-V fluorescent signals were measured with a confocal system (Zeiss LSM 700 META and LSM800).

### Immunofluorescence staining

After a 27-h or 44-h culture, cumulus cells were removed by repeated pipetting. Denuded oocytes were first fixed in 4% paraformaldehyde for 30 min and then permeabilized with 1% Triton X-100 at room temperature for at least 8 h. After blocking with 1% BSA-supplemented PBS for 1 h, the oocytes were incubated with a rabbit polyclonal anti-DRP1 antibody (1:100) at 4 °C overnight. After three washes in washing buffer (0.1% Tween 20 and 0.01% Triton X-100 in PBS), the oocytes were stained with a FITC-anti-rabbit IgG or TRITC-anti-rabbit IgG (1:100) antibodies for 1 h at room temperature. Oocytes were incubated for 1 h in α-tubulin-FITC and Phalloidin-TRITC stain, and then washed three times (2 min per wash) in PBS containing 0.1% Tween 20 and 0.01% Triton X-100. The samples were then co-stained with Hoechst 33342 (10 μg/mL in PBS) for 15 min. The oocytes were mounted on slides and examined with a confocal laser-scanning microscope (Zeiss LSM 700 META or LSM800). Each experiment was repeated at least three times, and at least 15 oocytes were examined.

### ImageJ analysis

We used ImageJ to analyze photographs of MI-stage oocytes after immunofluorescent staining to detect the changes in the mitochondrial, ROS, and annexin-V fluorescence signals collected by confocal laser-scanning microscopy (Zeiss LSM 700 META or LSM800). At least three replicates were tested for each control or treatment, and all replicates contained at least 10 samples. The averages of each group were analyzed by GraphPad Prism 5 (GraphPad Software Inc., La Jolla, CA, USA) and SPSS 12.0 statistical software (SPSS Inc., Chicago, IL, USA).

### Western blot analysis

To detect the levels of DRP1, 70 denuded porcine oocytes were collected after 27 and 44 h cultures, lysed in 12 μL of Laemmli sample buffer (SDS sample buffer containing 2-mercaptoethanol), boiled at 100 °C for 10 min, and immediately frozen at − 20 °C until use. The total protein in the 17 μL system described above underwent 10% sodium dodecyl sulfate-polyacrylamide gel electrophoresis (Invitrogen) for 1 h at 200 V and 4 °C, and the proteins were transferred to a polyvinylidene fluoride membrane (Invitrogen) for 1 h at 30 V and 4 °C. The membranes were blocked with 5% nonfat dry milk in Tris-buffered saline containing 0.1% Tween 20 for 1 h, and then incubated with anti-DRP1 and GAPDH antibodies overnight at 4 °C. After washing three times in Tris buffered saline with Tween (10 min each), the membranes were incubated at room temperature for 1 h with horseradish peroxidase-conjugated goat anti-rabbit IgG (1:1000; Thermo Fisher, Pittsburgh, PA, USA). Finally, the membranes were processed with high-sig ECL western blotting substrate (Tannon, Shanghai, China). Equal protein loading was confirmed by detecting GAPDH.

### Statistical analysis

At least three replicates of each treatment were tested, and the results are reported as mean ± standard error. The independent-sample *t*-test was used to compare data between the groups using GraphPad Prism 5 and SPSS 12.0 statistical software. Results were considered significant at *P* < 0.05.

## Results

### Expression and localization of DRP1 in porcine oocytes

**Fig. 1:**
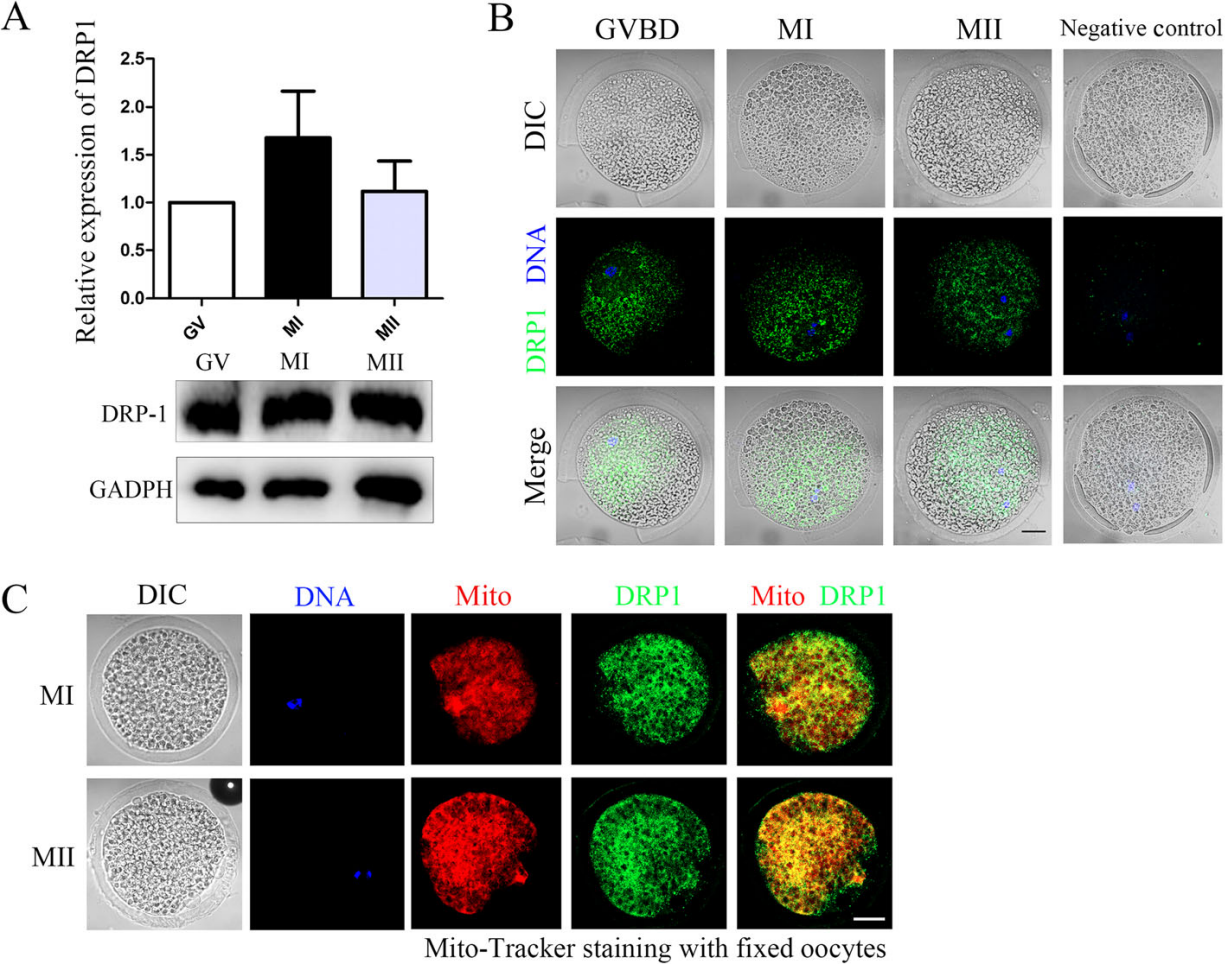
Expression and localization of dynamin-related protein 1 (DRP1) in porcine oocytes during maturation. **a** Western blot results for DRP1 protein expression. DRP1 was expressed throughout maturation of porcine oocytes. **b** Localization of DRP1 during porcine oocyte maturation. The z-stack scanning model was adopted to acquire the images. DRP1 localized in the cytoplasm at the germinal vesicle breakdown (GVBD), metaphase I (MI), and MII stages and DRP1 was asymmetrically distributed in porcine oocytes. Green, DRP1; blue, DNA. Bar = 20 μm. **c** Double staining of DRP1 and mitochondria show that DRP1 was co-localized with mitochondria in porcine oocytes. Green, DRP1; red, Mito-tracker; blue, DNA. Bar = 20 μm

We first analyzed the expression and localization of DRP1 during porcine oocyte meiotic maturation. As shown in Fig. 1a, the western blot results indicated that DRP1 was expressed at the GV, MI, and MII stages during porcine oocyte maturation. We also examined localization of DRP1 in porcine oocytes. The results showed that DRP1 was localized in the cytoplasm of porcine oocytes at the germinal vesicle breakdown (GVBD), MI, and MII stages (Fig. 1b), and that DRP1 distribution was asymmetric in the cytoplasm, with accumulated signals near the chromosomes. This localization pattern is similar to that of mitochondria in porcine oocytes. To confirm this pattern, we co-stained DRP1 with mitochondria in the oocytes at MI and MII stages. The results showed that the DRP1 signals overlapped with mitochondria, indicating that DRP1 co-localizes with mitochondria during porcine oocyte maturation (Fig. 1c).

### Inhibiting DRP1 activity results in failed porcine oocyte maturation

**Fig. 2:**
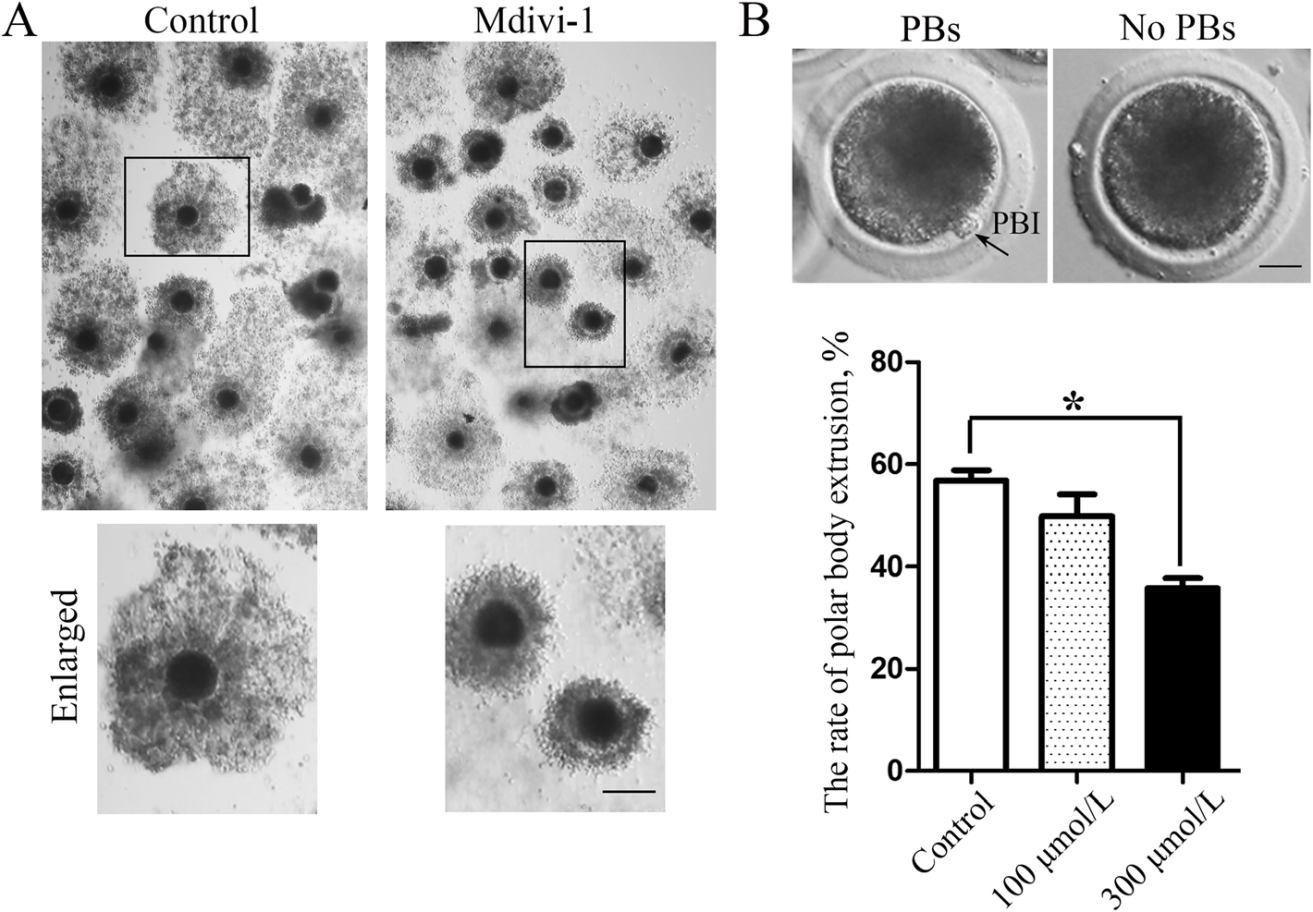
Inhibiting dynamin-related protein 1 (DRP1) activity resulted in failed porcine oocyte maturation. **a** After a 44-h treatment, the cumulus oocyte complex (COC) had expanded in the control group; however, failed COC expansion was observed after inhibiting DRP1. Bar = 100 μm. **b** Typical polar body extrusion and a statistical analysis of data for the rate of polar body extrusion. The rate of polar body extrusion was significantly lower in the DRP1 inhibited group than the control group. Bar = 20 μm

We explored the function of DRP1 during porcine oocyte maturation by treating oocytes with a non-competitive DRP1 inhibitor (Mdivi-1), which inhibits and inactivates circulating GTP and GDP. We analyzed COC expansion, as it is an important index of oocyte maturation. After a 44-h treatment with 300 μmol/L Mdivi-1, the COCs in the control group were well expanded, while the COCs showed expansion defects after inhibiting DRP1 activity (Fig. 2a). We then examined polar body extrusion after inhibiting DRP1 activity. The results showed that the 300 μmol/L Mdivi-1 treatment caused a significant decrease in polar body extrusion in porcine oocytes compared with the control group, while there was no difference between the control group and the 100 μmol/L treatment group (control group 56.87% ± 1.98%, oocyte number *n* = 80 vs. 100 μmol/L treatment group, 49.87 ± 4.21%, *n* = 80, *P* > 0.1; 300 μmol/L treatment group, 5.87% ± 1.88%, *n* = 80; *P* < 0.05; Fig. 2b). These data indicated that loss of DRP1 activity affects porcine oocyte maturation.

### Inhibiting DRP1 activity causes mitochondrial dysfunction in porcine oocytes

**Fig. 3:**
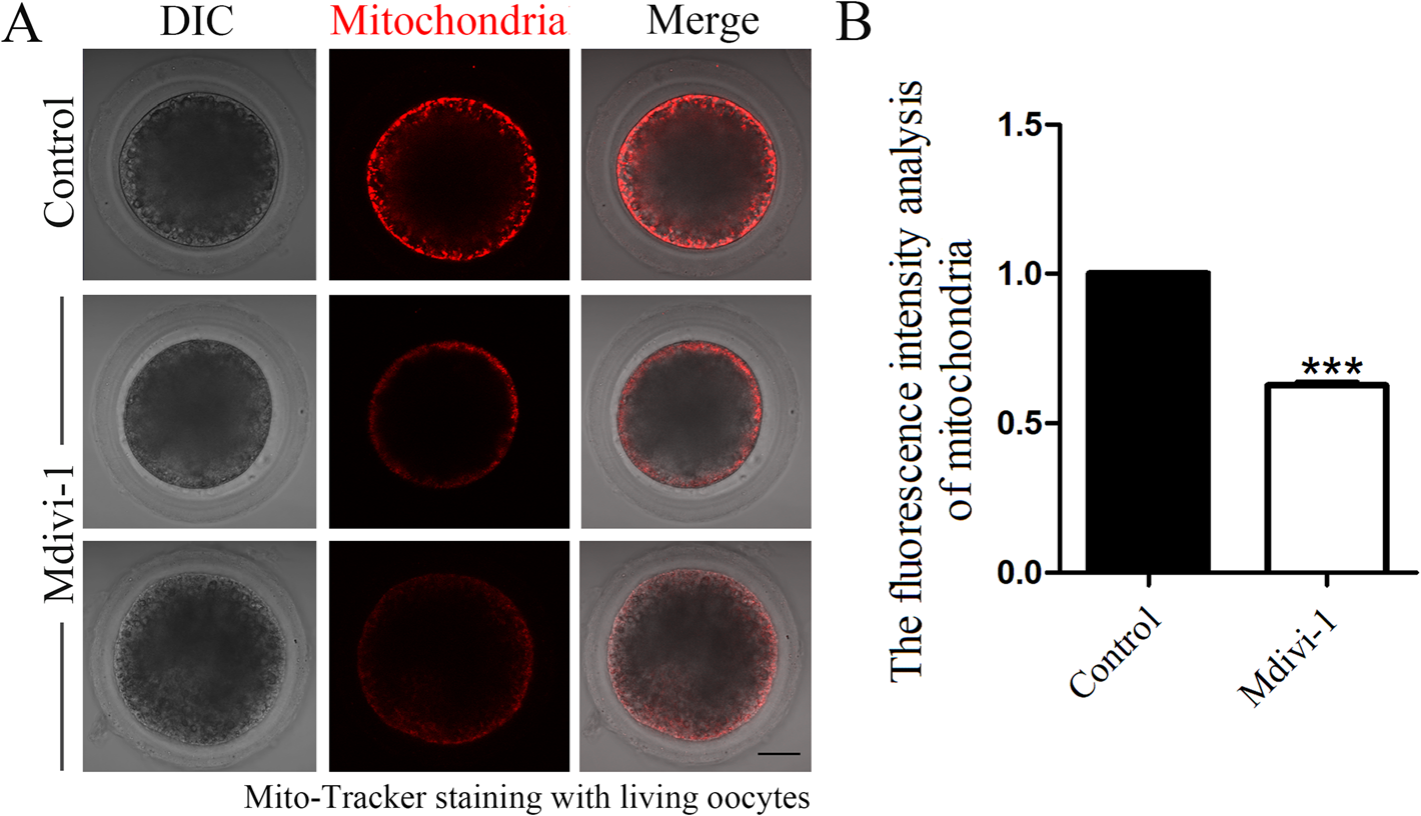
Mdivi-1 treatment inhibits mitochondrial aggregation in porcine oocytes. **a** Mito-tracker signals in the control and treatment group oocytes. Most control oocytes exhibited strong fluorescence signals, while the signals were much weaker in the treated oocytes. Red, Mito-tracker. Bar = 20 μm. **b** Mito-tracker fluorescence intensity analysis. Fluorescence intensity was much lower in the treatment group oocytes than the control oocytes

We next examined the porcine oocyte maturation defects caused by a DRP1 deficiency during mitochondrial fission. We cultured the oocytes for 27 h, the time point when most porcine oocytes reach the MI stage. Then, we detected the mitochondrial signal by Mito-tracker staining. As shown in Fig. 3a, mitochondria in most control oocytes exhibited strong signals and accumulated to the oocyte cortex, while the mitochondrial signals were much weaker in the oocytes of the DRP1 inhibited group. To assess the effect on the mitochondrial membrane potential after inhibiting DRP1, Mito-tracker fluorescent intensity was measured with ImageJ software. The treated oocytes were mounted on the same dish with control oocytes to standardize the fluorescence measurements. As shown in Fig. 3b, Mito-tracker fluorescence intensity of the treatment-group oocytes was significantly lower than that of the control group, which further confirmed our results (control group 1.0, *n* = 30 vs. treatment group 0.62 ± 0.01, *n* = 28; *P* < 0.05).

### Inhibiting DRP1 activity induces oxidative stress in porcine oocytes

**Fig. 4:**
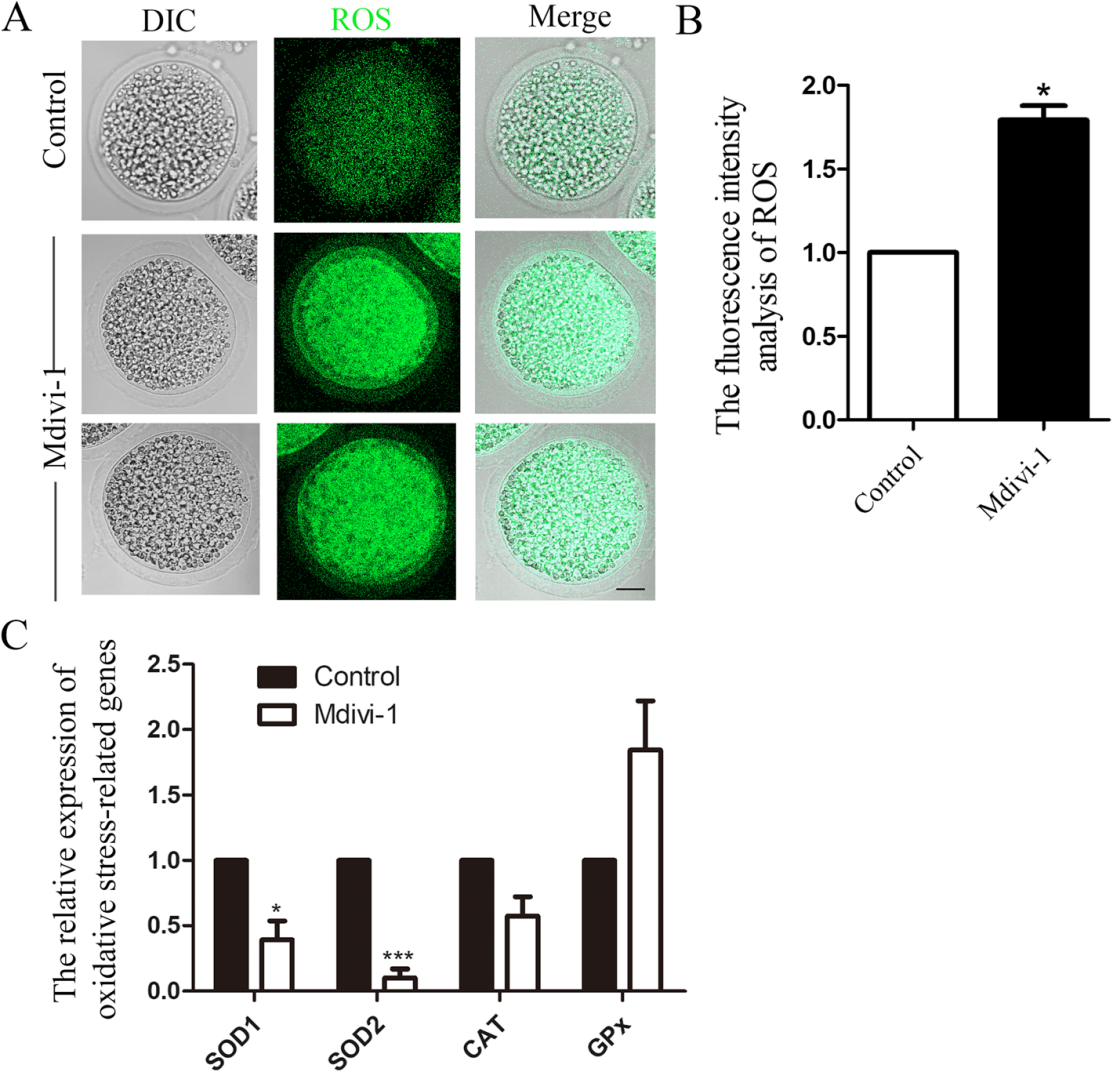
Mdivi-1 treatment induces oxidative stress in porcine oocytes. **a** The reactive oxygen species (ROS) level in the control and treatment groups of oocytes. Most control oocytes revealed weak ROS fluorescence signals, while the signals were much stronger in treated oocytes. Green, ROS. Bar = 20 μm. **b** ROS fluorescence intensity analysis. Fluorescence intensity was much higher in the treatment group oocytes than the control oocytes. **c** Superoxide dismutase (SOD)1 and SOD2 mRNA levels decreased significantly while the glutathione peroxidase (GPx) mRNA level increased significantly increased after inhibiting DRP1

Mitochondrial dysfunction can cause oxidative stress. We then examined whether a DRP1 deficiency induced oxidative stress in porcine oocytes. We first examined ROS levels, the main oxidative stress indicator. As shown in Fig. 4a, the ROS level was low in control oocytes; however, the DRP1 inhibited oocytes showed high intensity ROS signals, indicating the occurrence of oxidative stress after the DRP1 deficiency. The ROS fluorescence intensity analysis also confirmed this observation. The ROS fluorescence intensity in the treated oocytes was significantly higher than that in the control oocytes (control group 1.0, *n* = 30 vs. treatment group 1.79 ± 0.08, *n* = 28; *P* < 0.05; Fig. 4b). We also analyzed the expression of several oxidative stress-related genes. As shown in Fig. 4c, the expression levels of SOD1 and SOD2 decreased significantly, while GPx expression increased significantly, which further confirmed our results (SOD1: control group 1.0, n = 30 vs. treatment group 0.39 ± 0.14, *n* = 33; *P* < 0.05; SOD2: control group 1.0, *n* = 30 vs. treatment group 0.10 ± 0.07, *n* = 33; *P* < 0.05).

### Inhibiting DRP1 activity induces apoptosis in porcine oocytes

**Fig. 5:**
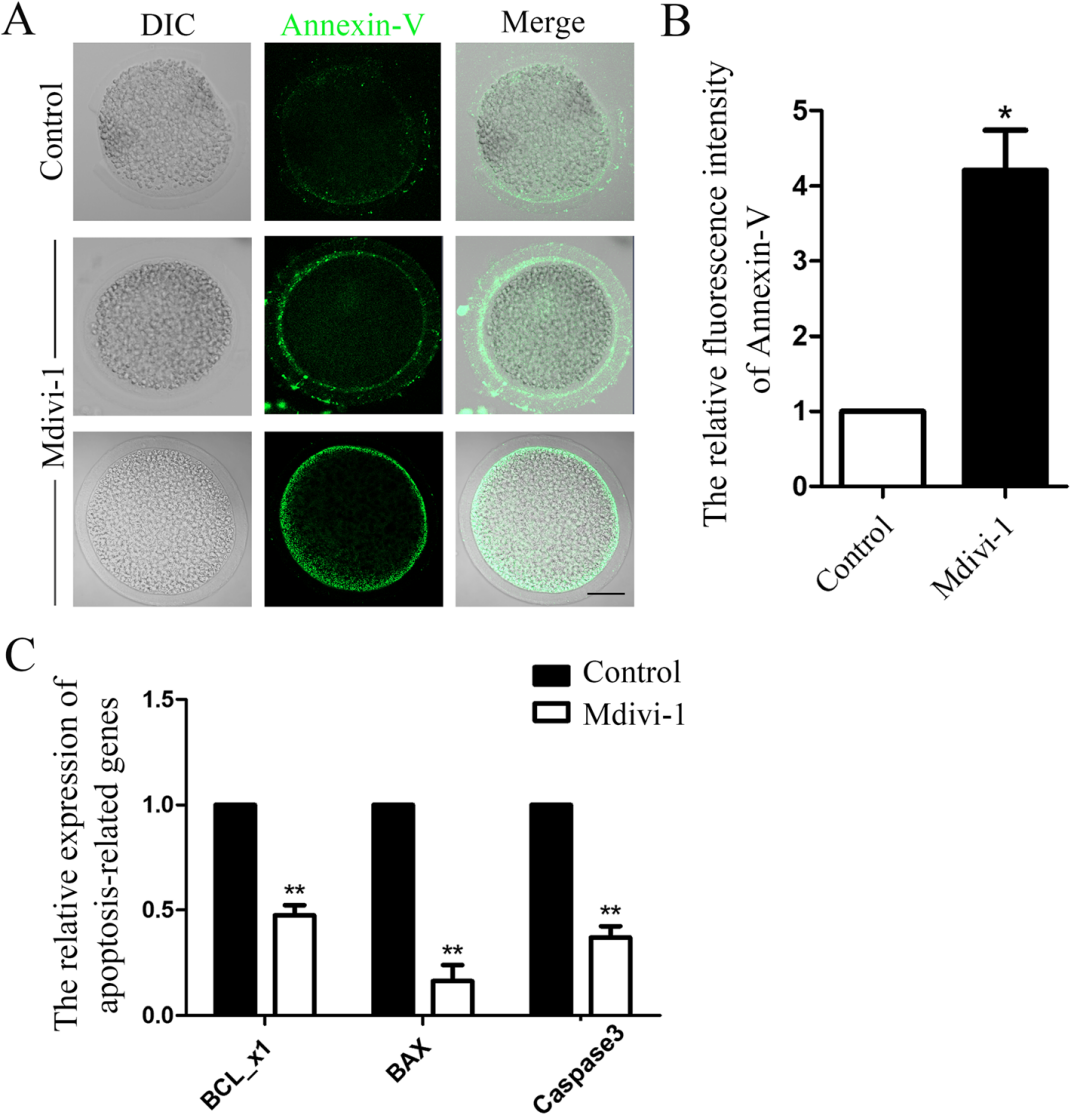
Mdivi-1 treatment induces apoptosis in porcine oocytes. **a** Annexin-V signals in the control and treatment group oocytes. Most control oocytes exhibited weak Annexin-V fluorescence signals, while the signals were much stronger in treated oocytes. Green, Annexin-V. Bar = 20 μm. **b** Annexin-V fluorescence intensity analysis. Fluorescence intensity was much higher in the treatment group oocytes than the control oocytes. **c** BCL_x1, BAX, and caspase3 mRNA levels decreased significantly after inhibiting DRP1 in porcine oocytes

Oxidative stress generally induces apoptosis. Thus, we examined whether a DRP1 deficiency could lead to the early stage of apoptosis in porcine oocytes. We first employed Annexin-V staining, an indicator of early apoptosis. As shown in Fig. 5a, very weak Annexin-V fluorescence signals originated in control oocytes. However, the DRP1 inhibited oocytes showed clear positive Annexin-V signals, indicating the occurrence of early apoptosis. The Annexin-V fluorescence intensity analysis also confirmed this finding. Annexin-V fluorescence intensity was significantly higher in treated oocytes than that in control oocytes (control group 1.0, *n* = 30 vs. treatment group 4.21 ± 0.53, *n* = 28; *P* < 0.05; Fig. 5b). We also analyzed the expression of several apoptosis-related genes. As shown in Fig. 5c, the expression of BCL_x1 and BAX and caspase3 decreased significantly, which further confirmed our results. (BCL_x1: control group 1.0, *n* = 30 vs. treatment group 0.47 ± 0.05, *n* = 32; *P* < 0.05; BAX: control group 1.0, *n* = 30 vs. treatment group 0.16 ± 0.07, *n* = 32; *P* < 0.05; caspase3: control group 1.0, *n* = 30 vs. treatment group 0.37 ± 0.05, *n* = 32; *P* < 0.05).

## Discussion

In this study we explored the expression and functions of DRP1 in porcine oocytes. We showed that inhibiting DRP1 activity affected porcine oocyte maturation, and loss of DRP1 activity disrupted mitochondrial function, which disturbed the ROS level and induced oxidative stress and apoptosis. These results indicate that DRP1 is an important factor during porcine oocyte maturation.

Our results show that DRP1 expression and localization in porcine oocytes was similar to mitochondria, as both DRP1 and mitochondria were distributed asymmetrically in the cytoplasm. This localization pattern was similar to previous reports in other models. A previous study showed that endogenous Drp1 localizes to mitochondria, which is consistent with a role in mitochondrial division [18]. Further studies have reported that DRP1 is located at the mitochondrial fission points and is involved in mitochondrial fission [10, 19]. One study illuminated the process of DRP1 localization more clearly that the DRP1 oligomers on the mitochondria progressively mature by incorporating smaller mitochondria-bound Drp1 units, and then the DRP1 oligomers are translocated directionally along the mitochondria [20]. Many of the Drp1-positive dots are localized to mitochondria in mouse oocytes [16]. Our results indicate the conserved localization of DRP1 in different models including mouse oocytes, and this DRP1 localization pattern in porcine oocytes indicates its relationship with mitochondria.

We then inhibited DRP1 activity to explore the roles of DRP1 in porcine oocytes. The results showed that loss of DRP1 activity induced aberrant cumulus cell expansion and polar body extrusion, two important indices of mammalian oocyte maturation, indicating the critical roles of DRP1 in porcine oocytes. As a fission factor of mitochondria, DRP1 is closely related to mitochondrial dynamics and arrangement [21, 22]. As DRP1 was co-localized with mitochondria in porcine oocytes, we first examined the mitochondria distribution. As results, DRP1 was essential for the energized state of mitochondria under the cortex of porcine oocytes. Similar DRP1 functions have been reported: reducing the amount of DRP1 that accumulates around mitochondria results in a delay of mitochondrial division in the U2OS cell line [20]. Moreover, DRP1 inhibits tumor cell growth by affecting mitochondrial function in brain tumor initiating cells (BTICs) [23]. The absence of Drp1 during Drosophila spermatogenesis leads to abnormal clustering of mitochondria in mature primary spermatocytes [24]. Moreover, a decreased number of neurites and defective synapses may be related with aggregated mitochondria in the NS-Drp1 (−/−) mouse forebrain [14]. Knockdown of DRP1 in mouse oocytes causes mitochondrial rearrangement [16]. Our results were consistent with these previous studies showing the roles of DRP1 in the mitochondrial dynamics of porcine oocytes.

We explored the possible causes for porcine oocyte maturation defects after DRP1 deficiency. Mitochondrial dysfunction is associated with oxidative stress, which is a common phenotype of aging, age-related diseases, and cancer [25]. The main cellular source of ROS is the mitochondrion [26]. Mitochondrial dysfunction can induce overproduction of ROS, leading to oxidative stress and damage to cells [27]. Therefore, we detected ROS levels after inhibiting DRP1, and the results indicated that a DRP1 deficiency caused overproduction of ROS and induced oxidative stress in porcine oocytes. Our findings verified the effects of DRP1 on oxidative stress in the porcine oocyte model. The roles of DRP1 on the induction of oxidative stress have been reported in many models: T-2 toxin treatment of human liver cells causes an increase in DRP1 expression which causes over-production of ROS [28]. MHY-1684 activates DRP1 and inhibits mitochondrial fusion in cardiac progenitor cells, which further induces oxidative stress [29]. Prx5 inhibits ERK-Drp1-induced mitochondrial fragmentation by regulating oxidative stress [30]. In addition, myocytes cultured with DHA have high ROS levels and downregulated DRP1 [31]. Dexamethasone increases ROS production and decreases the levels of mitochondrial fission proteins (Fis1 and DRP1) in human neuroblastoma cells [32]. These findings, together with our data, indicate the relationship between DRP1 and the regulation of ROS in different models.

Mitochondria play an important role in mammalian apoptotic cell death [33]. The level of intracellular ROS increases during the early stage of apoptosis [34]. The production of ROS is accompanied by apoptotic and autophagic cell death. Extensive ROS production triggers apoptosis and further affects oocyte maturation [35, 36]. Our results showed that loss of DRP1 activity induced early apoptosis in porcine oocytes. Similar results have been reported by other studies: Silencing Drp1/Parkin in liver cells significantly amplifies apoptotic signaling as demonstrated by cytochrome *c* release, caspase3 activity, and cleavage of poly (ADP-ribose) polymerase [37]. RhoA activates DRP1 through phosphorylation at Ser 616, protecting rat cardiomyocytes from cell death [38]. Targeting DRP1 in BTICs using RNA interference or pharmacologic inhibition induces apoptosis and inhibits tumor growth [23]. Moreover, increased DRP1-mediated mitotic fission results in decreased apoptosis in pulmonary artery smooth muscle cells [39]. These results are consistent with our findings and all show that DRP1 has a restraining effect on apoptosis in porcine oocytes.

## Conclusions

In conclusion, our results indicate that DRP1 is essential for porcine oocyte maturation through its effects on mitochondrial function, and that loss of DRP1 activity induces oxidative stress and apoptosis in porcine oocytes.

## Data Availability

All data generated or analyzed during this study are included in this published article.

## Abbreviations

COCs: Cumulus-oocyte complexs
ROS: Reactive oxygen species
MI: Metaphase I
MII: Metaphase II
GV: Germinal vesicle
GVBD: Germinal vesicle breakdown
ATI: Anphase/telophase I
